# Circulating CXCL10 and IL-6 in solid organ donors after brain death predict graft outcomes

**DOI:** 10.1038/s41598-021-86085-6

**Published:** 2021-03-23

**Authors:** Lorenzo Piemonti, Valeria Sordi, Silvia Pellegrini, Giulia Maria Scotti, Marina Scavini, Viviana Sioli, Andrea Gianelli Castiglione, Massimo Cardillo

**Affiliations:** 1 Diabetes Research Institute, IRCCS San Raffaele Scientific Institute, Via Olgettina 60, 20132 Milan, Italy; 2 Vita-Salute San Raffaele University, Milan, Italy; 3 Center for Translational Genomics and Bioinformatics, IRCCS San Raffaele Hospital, Milan, Italy; 4 Transplant Coordination Unit, Fondazione Ca’ Granda, Ospedale Maggiore Policlinico, Milan, Italy; 5 Azienda Ospedaliera S. Martino, Genoa, Italy

**Keywords:** Cytokines, Prognostic markers, Medical research

## Abstract

We tested the hypothesis that circulating CXCL10 and IL-6 in donor after brain death provide independent additional predictors of graft outcome. From January 1, 2010 to June 30, 2012 all donors after brain death managed by the NITp (n = 1100) were prospectively included in this study. CXCL10 and IL-6 were measured on serum collected for the crossmatch at the beginning of the observation period. Graft outcome in recipients who received kidney (n = 1325, follow-up 4.9 years), liver (n = 815, follow-up 4.3 years) and heart (n = 272, follow-up 5 years) was evaluated. Both CXCL-10 and IL-6 showed increased concentration in donors after brain death. The intensive care unit stay, the hemodynamic instability, the cause of death, the presence of risk factors for cardiovascular disease and the presence of ongoing infection resulted as significant determinants of IL-6 and CXCL10 donor concentrations. Both cytokines resulted as independent predictors of Immediate Graft Function. Donor IL-6 or CXCL10 were associated with graft failure after liver transplant, and acted as predictors of recipient survival after kidney, liver and heart transplantation. Serum donor IL-6 and CXCL10 concentration can provide independent incremental prediction of graft outcome among recipients followed according to standard clinical practice.

## Introduction

Success of organ transplantation from deceased donor in the short term has progressively improved, with 1-year allograft survival of ~ 95%, ~ 85% and ~ 85% for kidney, liver and heart transplant, respectively^1–3^. Unfortunately, the ultimate goal of providing long-term graft survival has not been achieved, with a relatively stable rate of attrition, with a 5-year allograft survival of ~ 85%, ~ 75% and ~ 75% for kidney, liver and heart transplant, respectively^4–6^, i.e., 15–25% of graft loss within 5 years after transplantation. Whereas early immune-mediated injury is primarily responsible for graft dysfunction and failure, the influence of antigen-independent events may have been underestimated. This concept is supported by data showing similar survival rates for kidneys from living-unrelated donors and one-haplotype matched living-related donors^7–9^. Furthermore, organs from living donors, regardless of their relatedness to recipients, have consistently superior outcomes than those from donor after brain death^7,10,11^. An obvious difference between living and donor after brain death are the potential effects of brain death. During and after brain death, circulating leukocyte traffic through peripheral organs slows, and cells adhere to the vascular endothelium and infiltrate the tissues^12^. As a consequence, donor brain death promptly upregulates inflammatory mediators in peripheral organs with massive increase of major histocompatibility antigens, adhesion molecules, cytokines, and other acute-phase proteins^13–16^. This, in turn, may amplify host alloresponsiveness both early after transplant and in the long-term. The different cellular and molecular changes presumably occur secondary to the initial activity of catecholamines^17,18^ as well as circulatory cytokines originating from the injured brain and activation of systemic host responses^19–21^. As a result, organs from donors after brain death that are transplanted into unmodified allogeneic hosts experience function loss at an accelerated rate compared to those from living donors^22,23^. This concept is supported by the clinical finding of a consistently inferior outcome (i.e., function and survival) of kidney allografts with delayed function plus acute rejection compared to organs with a single insult or no insult at all^24–27^. However, there is still a gap in knowledge regarding the specific pathways associated with inferior post-transplant outcomes^28^. The role of the “immune memory” of the transplanted organ in triggering the host immune response and the mediators involved in this process are still not fully elucidated^15,20,29–37^. Here, a prospective observational study to assess the predictive value of circulating CXCL10 and IL-6 in the donor after brain death for graft survival and function following allotransplantation is presented. We focused our analysis on these two immunological mediators because they have a common double advantage: to be extremely relevant for the immune response after transplantation and to be the target of drugs already available on the market (i.e., Tocilizumab, Sarilumab) or in advanced experimental clinical phases in humans (i.e., Eldelumab) (45)(46).

## Results

### Study cohort

From January 1, 2010 to June 30, 2012 1100 donors after brain death were prospectively included in the study: 533 (48.5%) from Lombardia, 78 (7.1%) from Liguria, 270 (24.5%) from Veneto, 87 (7.9%) from Friuli‐Venezia Giulia, 102 (9.3%) from Marche and 30 (2.7%) from the Autonomous Province of Trento. During the same period, 2869 patients underwent various types of transplant in 21 different centres, receiving organ from 1074 out of 1100 donors included in the study (see Supplementary Table S1). Among the different types of transplant, kidney transplant accounted for the highest patient group (single n = 1325, 46.2%; double n = 150; 5.2%), followed by liver (whole n = 815, 28.4%; right lobe n = 56, 1.9%; left lobe n = 43, 1.5%), heart (n = 272, 9.4%), lung (double n = 82, 2.8%; single n = 35, 1.2%) and pancreas (alone n = 16, 0.5%; with kidney n = 46, 1.6%). The follow up study was performed for single kidney, whole liver and heart transplantation. The median follow up was 4.9 years (4.8–5; 95% CI) for kidney, 4.3 years for liver (4.2–4.5; 95% CI) and 5 years (4.9–5.1; 95% CI) for heart. Fourteen out of 1325 (1.1%), 2 out of 815 (0.24%) and 7 out of 272 (2.6%) patients receiving kidney, liver and heart transplant, respectively, were lost to follow up. Graft and patient survival are presented in Supplementary Figure S1.

### Elevated levels of cytokines in donor after brain death

**Figure 1:**
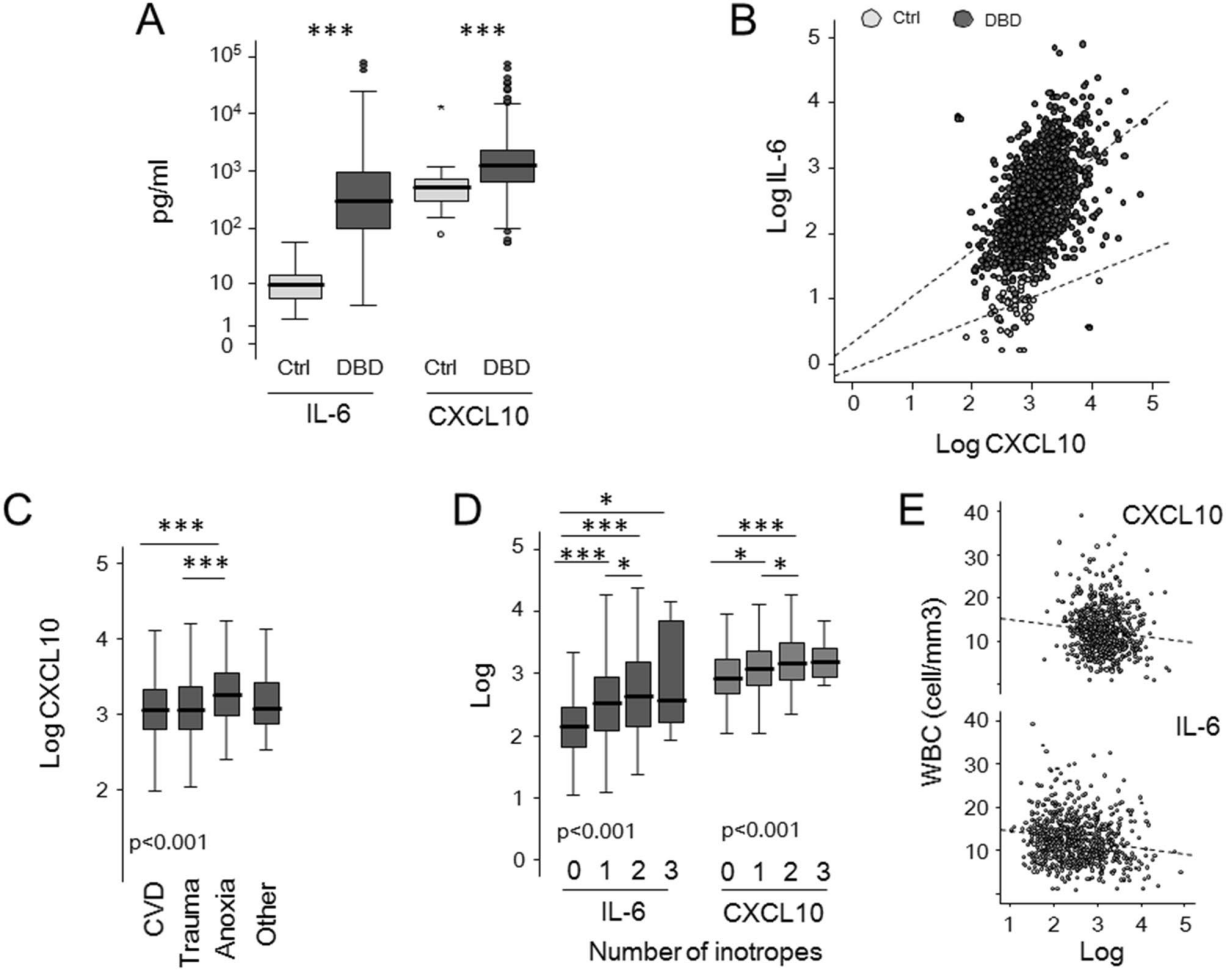
Circulating levels of IL-6 and CXCL10 in deceased donors and healthy subjects. (**A**) Circulating levels of IL-6 and CXCL10 in serum from healthy subjects (Ctrl, n = 55), and deceased donors (donation after brain death, DBD, n = 1100) *were* evaluated. The boxes represent the interquartile range, the line represents the median, and whiskers indicate the range of observed responses. *p < 0.05; **p < 0.01; ***< 0.001; Mann–Whitney U test. (**B**) Scatter plot illustrating the relationship between IL-6 and CXCL10. (**C**,**D**) Circulating levels of IL-6 and CXCL10 according to death cause and number of donor inotropes. The boxes represent the interquartile range, the line represents the median, and whiskers indicate the range of observed responses. P value was calculated by Kruskal–Wallis test; *< 0.05; ** p< 0.01; *** p< 0.001 at post-hoc analyses by Mann–Whitney U tests, p value adjusted for the number of comparisons done. (**E**) Scatter plot illustrating the relationship between IL-6, CXCL10 and white blood cell (WBC).

We tested circulating levels of CXCL10 and IL-6 in 1100 donors after brain death and 55 heathy subjects (Fig. 1A). Compared with healthy control sera, donor after brain death sera contained significantly higher levels of IL-6 [median 297 pg/ml (IQR 101–934) vs 9.7 pg/ml (IQR 5.2–14.5), p < 0.0001] and CXCL10 [median 1220 pg/ml (IQR 633–2,286) vs 512 pg/ml (IQR 311–752), p < 0.0001]. A significant correlation between the two cytokines was evident both in control (ρ 0.354; p = 0.008) and in donor sera (ρ 0.499; p < 0.001) (Fig. 1B). IL-6 and CXCL10 levels were similar between donors with organs deemed unsuitable (26 out 1100) or suitable for transplantation (data not shown).

### IL-6 and CXCL10 associate with multiple baseline donor characteristics

We analysed which of the available baseline donor characteristics were associated with the circulating IL-6 and CXCL10 (Table 1). Donor characteristics were grouped arbitrarily into five categories: demographic and health history, causes of death, variables related to intensive care duration and hemodynamic stability, biochemistry blood tests, infectious disease data (Table 1). Among demographic and health history, the presence of risk factors for cardiovascular disease (i.e., history of diabetes, hypertension and cardiopathy) and age, showed variable levels of positive correlation with both cytokines. CXCL10, but not IL-6, had significant association with the cause of death with higher level in donors deceased for anoxic events (Fig. 1C). Both cytokines had at least one significant correlation with variables related to intensive care duration and hemodynamic stability. The use of inotropes, in particular norepinephrine, was generally associated with higher levels of circulating CXCL10 and IL-6 (Fig. 1D). As expected, biochemistry blood tests showed some positive correlations with the two circulating cytokines, in particular creatinine, bilirubin and standardized prothrombin time. Of note, the two cytokines showed a negative correlation with the concentration of white blood cells (Fig. 1E). Among infectious disease data, donor blood/urine infections and positivity for CMV IgM showed a positive association with the two cytokines. We used a stepwise method for the variable selection to study which of the baseline donor characteristics explained most of the variance in IL-6 and CXCL10 levels. We included for each cytokine the variable resulted significant in univariate analysis. Collectively, baseline donor characteristics explained 14.4% (p < 0.001) and 12.1% (p < 0.001) of the variance in IL-6 and CXCL10 levels, respectively. Independent baseline donor characteristics explaining the variance in IL-6 values were the number of inotropes and vasopressors administered (R2 change: 0.07, p < 0.001), the presence of donor blood infection (R2 change: 0.038, p = 0.004), and WBC (R2 change: 0.036, p = 0.005). Anoxic event as cause of death (R2 change: 0.042, p = 0.004), total bilirubin concentration (R2 change: 0.031, p = 0.011), the presence of donor blood infection (R2 change: 0.026, p = 0.018) and the number of inotropes and vasopressors administered (R2 change: 0.021, p = 0.031) resulted the independent donor baseline characteristics explaining the variance in CXCL10 levels.

**Table 1:** Baseline donor characteristics and circulating levels of IL-6 and CXCL10.

Variable	IL-6			CXCL10		
	Median (IQR)		p	Median (IQR)		p
	Yes	No		Yes	No	
**Demographic and health history**						
Age	ρ^a^ = 0.136		** < 0.001**	ρ = 0.077		**0.01**
Male	338 (109–973)	259 (93–846)	0.073	1,277 (662–2,392)	1,148 (615–2,207)	0.208
Weight	ρ = 0.034		0.256	ρ = 0.03		0.322
BMI	ρ = 0.031		0.310	ρ = 0.008		0.792
Diabetes	369 (126–1075)	267 (100–854)	**0.033**	1241 (660–2384)	1189 (622–2273)	0.695
Hypertension	328 (120–10,779	254 (87–776)	**0.007**	1242 (680–2382)	1143 (585–2204)	**0.049**
Cardiomyopathy	339 (134–1017)	250 (91–930)	**0.017**	1429 (758–2519)	1022 (534–2077)	** < 0.001**
Cancer	358 (155–1443)	286 (102–841)	0.067	1561 (714–3222)	1155 (625–2252)	0.084
Smoke	1118 (603–2108)	1219 (652–2276)	0.362	268 (89–836)	289 (114–871)	0.116
Alcohol abuse	1317 (648–2752)	1160 (621–2228)	0.373	372 (136–953)	271 (101–834)	0.761
**Cause of death**						
Cause of death: cardiovascular	274 (97–964)	325 (105–875)	0.651	1144 (615–2145)	1325 (700–2583)	**0.015**
Cause of death: trauma	362 (124–851)	271 (93–942)	0.181	1125 (616–2264)	1227 (641–2284)	0.433
Cause of death: anoxic event	282 (69–911)	296 (103–934)	0.381	1809 (933–3457)	1149 (619–2222)	** < 0.001**
Cause of death: brain tumour	482 (76–1856)	293 (101–932)	0.643	1549 (637–3770)	1219 (634–2284)	0.494
Cause of death: others	242 (96–784)	302 (102–942)	0.684	1154 (710–2687)	1222 (630–2268)	0.631
**Intensive care duration and hemodynamic stability**						
Lengths of stay in ICU	ρ = 0.02		0.507	ρ = 0.251		** < 0.001**
Any inotrope administered	341 (118–99)	140 (64–287)	** < 0.001**	1235 (660–2372)	846 (480–1701)	** < 0.001**
Two or more inotropes	402 (140–1603)	264 (97–788)	** < 0.001**	140 (799–3133)	1132 (599–2174)	**0.001**
Number of inotropes	ρ = 0.204		** < 0.001**	ρ = 0.153		** < 0.001**
Norepinephrine administered	359 (124–1094)	223 (91–596)	** < 0.001**	1237 (662–2324)	1115 (577–2118)	0.085
Dopamine administered	307 (112–819)	275 (99–950)	0.472	1224 (666–2443)	1159 (596–2219)	0.111
Cardiac arrest	272 (103–875)	276 (103–875)	0.696	1324 (686–2475)	1100 (609–2183)	**0.019**
**Biochemistry blood tests**						
Blood type A	290 (102–791)	299 (101–1,017)	0.437	1,149 (665–2259)	1,242 (622–2333)	0.743
Blood type B	274 (118–777)	302 (100–933)	0.959	1104 (619–1788)	1233 (634–2324)	0.222
Blood type AB	185 (95–651)	302 (102–935)	0.394	1237 (641–3081)	1219 (630–2264)	0.698
Blood type 0	322 (95–1114)	280 (102–789)	0.259	1292 (615–2363)	1149 (653–2239)	0.385
Proteinuria during admission	292 (112–727)	313 (107–953)	0.96	1237 (656–2475)	1154 (613–2188)	0.199
Final WBC	ρ = -0.161		** < 0.001**	ρ = -0.097		**0.006**
Final Hb	ρ = 0.001		0.976	ρ = -0.031		0.393
Final creatinine	ρ = 0.194		** < 0.001**	ρ = 0.21		** < 0.001**
Final blood urea nitrogen	ρ = 0.055		0.151	ρ = 0.179		** < 0.001**
Final AST	ρ = -0.055		0.126	ρ = 0.051		0.158
Final ALT	ρ = -0.091		0.087	ρ = 0.068		0.057
Total bilirubin	ρ = 0.164		** < 0.001**	ρ = 0.131		** < 0.001**
Final GGT	ρ = -0.045		0.226	ρ = 0.138		** < 0.001**
Final amylase	ρ = 0.103		**0.010**	ρ = 0.035		0.381
Final international normalized ratio	ρ = 0.166		** < 0.001**	ρ = 0.163		** < 0.001**
**Infectious disease data**						
Donor urinary infection	404 (117–1268)	263 (92–841)	**0.030**	404 (117–1268)	263 (92–841)	**0.040**
Donor blood infection	341 (101–2086)	270 (95–822)	**0.039**	1509 (853–3063)	1152 (615–2142)	**0.003**
Donor pulmonary infection	311 (102–993)	251 (88–803)	0.107	1242 (641–2168)	1104 (609–2150)	0.221
HBcAb positive	324 (118–866)	287 (97–946)	0.419	1219 (656–2251)	1221 (631–2291)	0.803
HCV positive	304 (83–1279)	296 (102–932)	0.867	1527 (814–4412)	1208 (628–2259)	0.072
EBV-EBNA IgG positive	312 (103–944)	235 (80–717)	0.193	1228 (634–2333)	1043 (622–2039)	0.181
EBV-VCA IgG positive	310 (103–950)	203 (82–580)	**0.045**	1221 (633–2288)	1086 (619–2351)	0.520
CMV-IgG positive	311 (104–951)	229 (83–690)	0.057	1219 (634–2294)	1265 (604–2264)	0.602
CMV-IgM positive	3346 (2590–4103)	290 (101–932)	**0.037**	3110 (2204–4017)	1203 (633–2272)	0.127
HSV-1 IgG positive	287 (102–942)	312 (82–788)	0.639	1221 (632–2321)	1272 (649–1978)	0.597
HSV-2 IgG positive	229 (90–731)	311 (102–946)	0.205	1179 (646–2247)	1219 (632–2291)	0.6
Toxo-IgG positive	270 (92–797)	323 (111–973)	0.079	1222 (640–2316)	1162 (610–2233)	0.697
Toxo-IgM positive	323 (66–503)	297 (102–934)	0.673	1365 (847–2610)	1208 (633–2284)	0.567
TPHA positive	149 (83–864)	299 (102–934)	0.492	839 (523–2291)	1221 (634–2291)	0.309
VZV-IgG positive	299 (102–926)	288 (86–989)	0.885	1220 (622–2305)	1187 (703–2111)	0.954

^a^Spearman's Rank correlation coefficient.

Bold values indicate Statistically significant.

### Donor IL-6 and CXCL10 are independent negative predictors of immediate graft function (IGF)

**Figure 2:**
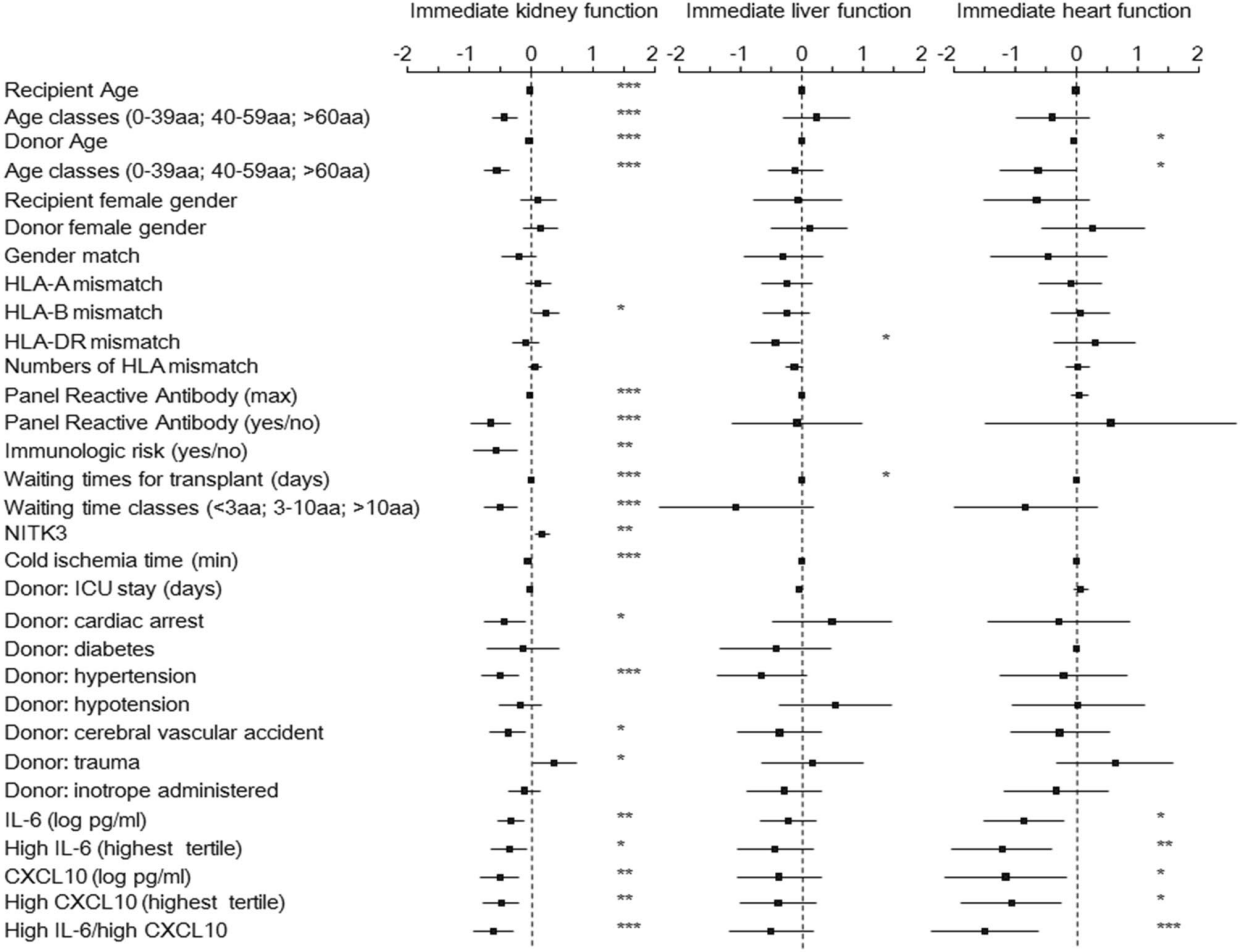
Univariate odd ratios for immediate graft function (IGF). The association between donor IL-6 and CXCL10, baseline variables and immediate graft function was assessed by a logistic regression analysis including patients receiving kidney, liver or heart transplant. All analyzed variables are presented. Dots represent the odds ratio (OR) after natural log transformation, lines the 95% confidence intervals. *p < 0.05; **p < 0.01; ***p < 0.001.

The association of donor IL-6 and CXCL10 levels with the IGF was performed using Logistic Regression analysis. For the regression models, cytokine concentrations were used as continuous variables after Log transformation and as binary variables (see “Method”***). In addition to the two cytokines, the following variables were included in the model: donor sex and age, recipient sex and age, donor cause of death (cardiovascular accident, trauma), donor characteristics (ICU stay, diabetes, hypertension, hypotension, cardiac arrest, cold ischemia time), donor inotrope administered (none, one, two or more), time spent on the waiting list, number of HLA mismatches (HLA-A, -B, and -DR antigens), maximum panel-reactive antibody (PRA) level, immunologic risk and NITK3^38^ (for kidney transplant only). The level of function of a graft in the immediate postoperative period was described to be correlated with long-term graft and patient survival. Confirming what is expected, even in our cohort the absence of IGF in the postoperative period was correlated with graft and recipient survival (Supplementary Figure S2): hazard ratios for graft failure were 3.1 (2.2–4.5; p < 0.001), 13 (8.4–20.2, p < 0.001) and 6.1 (3–13, p < 0.001) for kidney, liver and heart transplants, respectively. Concordantly, hazard ratios for recipient death were 1.6 (1.2–2.4, p = 0.049), 4.8 (2.9–8, p < 0.001) and 3.2 (1.7–5.9, p < 0.001) for kidney, liver and heart transplants, respectively. In the univariate analysis, we found a negative association between IL-6 or CXCL10 donor concentrations and IGF in kidney, heart but not liver recipients (Fig. 2). The multivariate analysis confirmed CXCL10 donor concentration as independent negative predictors of IGF in kidney recipient (Table 2) and IL-6 at the limit of the significance. Concordantly, high CXCL10 [OR 0.58 (0.4–0.84); p = 0.004] and high IL-6/CXCL10 [OR 0.59 (0.39–0.87); p = 0.011] categories were significantly associated with lower probability of immediate kidney function, while high IL-6 category showed a weak trend [OR 0.75 (0.51–1.09); p = 0.131]. In heart recipient, both CXCL10 and IL-6 donor concentration resulted as independent negative predictor of IGF (Table 2) and high IL-6 [OR 0.30 (0.13–0.68); p = 0.004], high CXCL10 [OR 0.31 (0.13–0.71); p = 0.006] and high IL-6/CXCL10 [OR 0.19 (0.08–0.48); p < 0.001] categories were all significantly associated with lower probability of immediate heart function.

**Table 2:** Logistic regression models of the predictors of IGF by multivariate analysis.

	IL-6		CXCL10	
	Odds ratio (95% CI)	p	Odds ratio (95% CI)	p
**Kidney transplant**				
Recipient age	0.99 (0.97–1.01)	0.42	0.99 (0.97–1.01)	0.39
Donor age	0.98 (0.96–1)	0.055	0.98 (0.96–1)	0.062
HLA-B mismatch	1.36 (1.01–1.82)	0.039	1.35 (1–1.8)	0.046
Panel reactive antibody (max)	0.99 (0.98–1.00)	0.325	0.99 (0.95–19	0.235
Day in waiting list	1 (0.99–1)	0.001	1 (0.99–1)	0.001
Cold ischemia time	0.97 (0.94–1)	0.047	0.97 (0.94–0.99)	0.043
Donor: cardiac arrest	0.45 (0.29–0.71)	0.001	0.47 (0.3–0.74)	0.001
Donor: hypertension	0.77 (0.52–1.13)	0.18	0.76 (0.51–1.12)	0.165
Donor: cerebral vascular accident	0.58 (0.33–0.99)	0.047	0.53 (0.-0.92)	0.024
Donor: trauma	0.67 (0.35–1.32)	0.25	0.59 (0.3–1.2)	0.14
Donor cytokine (log pg/ml)	0.76 (0.58–1)	0.055	0.55 (0.36–0.84)	0.006
**Liver transplant**				
HLA-DR mismatch	0.63 (0.42–0.96)	0.032	0.63 (0.42–0.96)	0.032
Waiting times for transplant (days)	0.99 (0.99–1)	0.019	0.99 (0.99–1)	0.017
Donor cytokine (log pg/ml)	0.82 (0.51–1.32)	0.42	0.69 (0.34–1.4)	0.30
**Heart transplant**				
Donor age	0.96 (0.92–0.99)	0.011	0.95 (0.92–0.99)	0.007
Donor cytokine (log pg/ml)	0.40 (0.20–0.81)	0.01	0.26 (0.09–0.76)	0.014

For each cytokine, donor and recipient characteristics with p values < 0.05 in univariate analysis were included in the multivariate analysis.

### IL-6 and CXCL10 can predict the long-term transplant outcome

**Figure 3:**
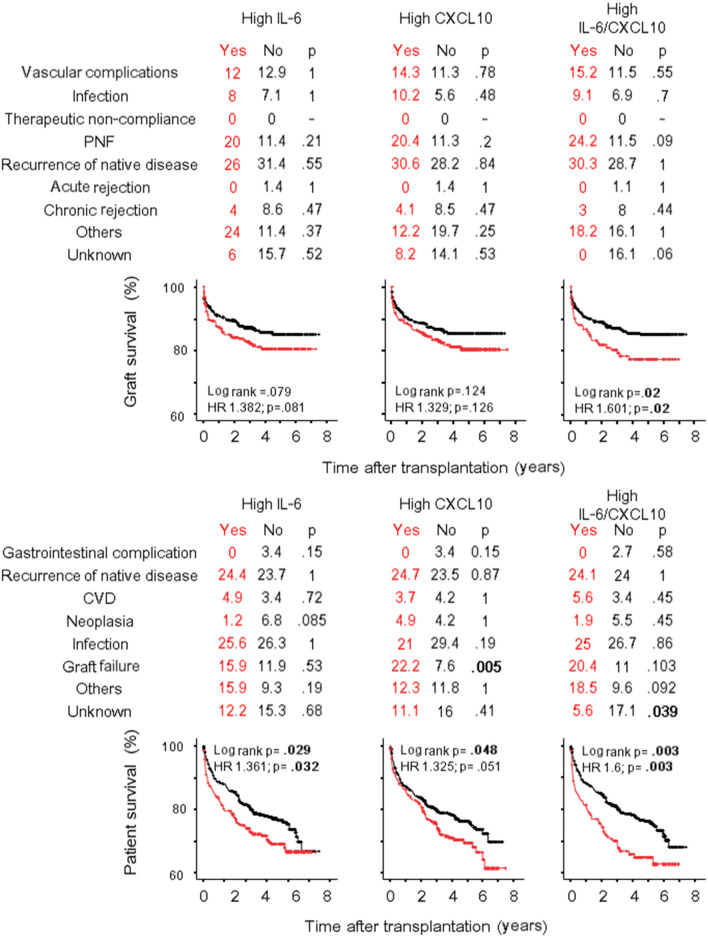
Graft and recipient survival after liver transplantation. Causes of graft failure (%), causes of recipient death (%) and Kaplan–Meier graft and recipient survival curves after liver transplant are represented according to high IL-6 and high CXCL10 categories. *PNF* primary non function, *CVD* cardiovascular disease.

**Figure 4:**
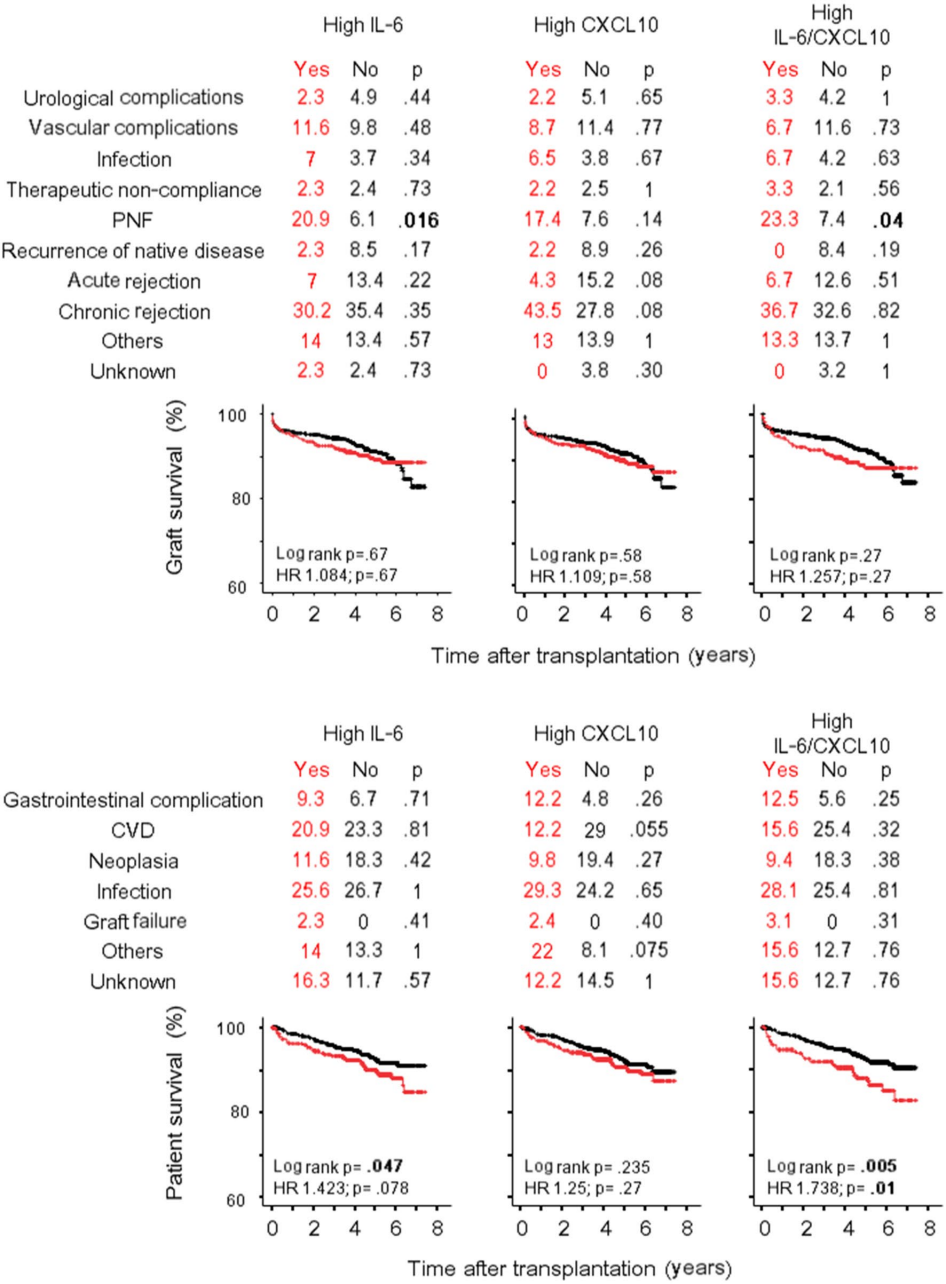
Graft and recipient survival after kidney transplantation. Causes of graft failure (%), causes of recipient death (%) and Kaplan–Meier graft and recipient survival curves after kidney transplant are represented according to high IL-6 and high CXCL10 categories. *PNF* primary non function, *CVD* cardiovascular disease.

**Figure 5:**
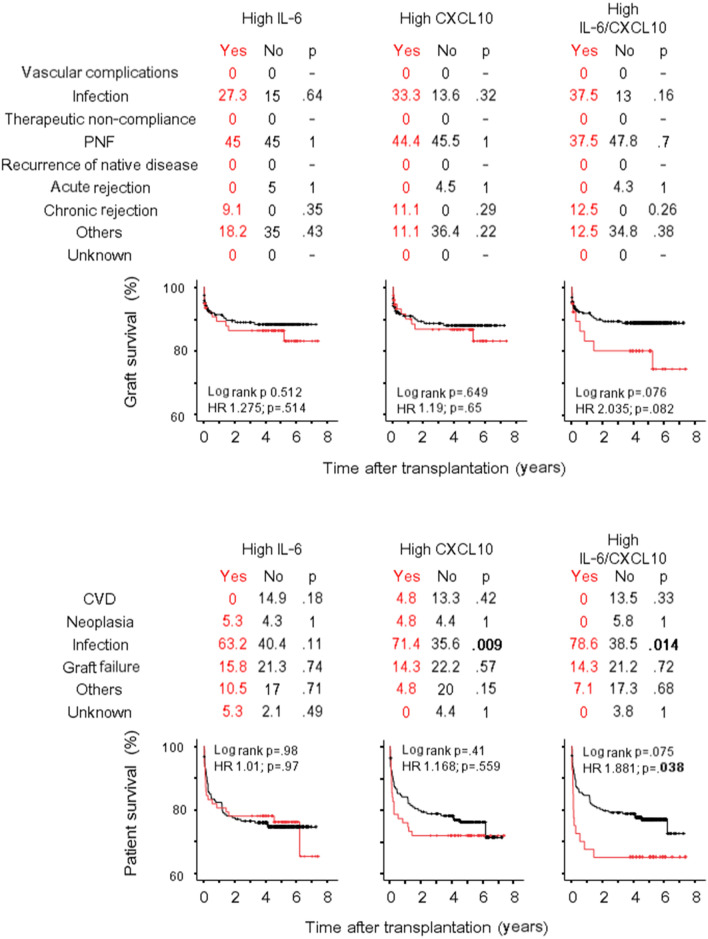
Graft and recipient survival after heart transplantation. Causes of graft failure (%), causes of recipient death (%) and Kaplan–Meier graft and recipient survival curves after heart transplant are represented according to high IL-6 and high CXCL10 categories. *PGF* primary graft failure, *CVD* cardiovascular disease.

The association of donor IL-6 and CXCL10 levels with the recipient and graft survivals was performed by Cox Regression analysis including variables as for IGF analysis. In univariate analysis (Supplementary Table S3), higher concentrations of donor IL-6 and CXCL10 were associated with both graft failure and recipient survival after liver transplant. The multivariate analysis (Table 3) confirmed both cytokines as independent predictors of liver failure [IL-6: HR 1.43 (1.09–1.88), p = 0.01; CXCL10: 1.5 (1–2.24), p = 0.05] and liver recipient survival [IL-6: HR 1.36 (1.06–1.75), p = 0.016; CXCL10: 1.45 (1–2.1), p = 0.052]. Causes of liver failure, causes of recipient death and Kaplan–Meier liver and recipient survival curves according to high IL-6 and high CXCL10 categories are represented in Fig. 3. Trends for a higher percentage of primary non-function as cause of liver failure, and a higher percentage of graft failure as cause of liver recipient death were evident, especially when the high IL-6/CXCL10 category was analysed. Notably, in contrast to liver, less consistent results have been obtained for kidney and heart transplantation. In fact, IL-6 and CXCL10 were not associated with kidney failure in univariate or multivariate analysis (Table 3, Supplementary Table S3) even if the prevalence of primary no function was significantly higher in high IL-6 category (Fig. 4) and a trend toward a higher percentage of chronic rejection (but not acute rejection) was evident in the high CXCL10 category. After heart transplant IL-6 and CXCL10 concentration were not associated with graft survival (Table 3; Supplementary Table S3), but a trend was evident when the high IL-6/CXCL10 category was considered in the analysis [univariate: HR 2.04 (0.91–4.53), p = 0.08; multivariate: 2.15 (0.96–4.8), p = 0.064] (Fig. 5). High IL-6/CXCL10 category was significantly inversely associated with recipient survival after both kidney (uni- and multivariate analyses) and heart transplantation (univariate analysis). Among death causes, a higher percentage of infections as cause of death was observed after heart transplant in high IL-6/CXCL10 category.

**Table 3:** Cox regression models of the predictors of graft failure and recipient death by multivariate analysis.

	Graft failure				Recipient death			
	IL-6		CXCL10		IL-6		CXCL10	
	Hazard ratio (95% CI)	p	Hazard ratio (95% CI)	p	Hazard ratio (95% CI)	p	Hazard ratio (95% CI)	p
**Kidney transplant**								
Donor age	1.01 (0.99–1.03)	0.253	1.01 (0.99–1.03)	0.253	1.04 (1.03–1.06)	< 0.001	1.05 (1.03–1.06)	< 0.001
Panel Reactive Antibody (max)	1.01 (0.99–1.02)	0.201	1.01 (0.99–1.02)	0.203	1.01 (0.99–1.02)	0.480	1.01 (0.99–1.02)	0.480
Immunologic risk (yes/no)	0.61 (0.17–2.13)	0.436	0.61 (0.17–2.13)	0.437	1.35 (0.62–2.91)	0.473	1.32 (0.61–2.85)	0.473
NITK3	0.79 (0.53–1.16)	0.229	0.79 (0.53–1.16)	0.229	–		–	
Cold ischemia time (min)	1.02 (0.98–1.06)	0.241	1.02 (0.98–1.06)	0.241	–		–	
Donor: hypertension	1.43 (0.91–2.24)	0.120	1.43 (0.91–2.23)	0.120	1.34 (0.86–2.08)	0.182	1.35 (0.87–2.01)	0.182
Donor: cerebral vascular accident	1.55 (0.96–2.5)	0.074	1.55 (0.96–2.5)	0.075	–		–	
Donor: inotrope administered	1.36 (0.95–1.94)	0.097	1.36 (0.95–1.95)	0.097	–		–	
Donor cytokine (log pg/ml)	0.99 (0.71–1.37)	0.936	0.99 (0.71–1.37)	0.934	1.18 (0.87–1.61)	0.29	0.93 (0.57–1.5)	0.76
High IL-6/CXCL10^a^	0.976 (0.59–1.62)			0.926	1.604 (1.019–2.525)			0.041
**Liver transplant**								
Recipient age	0.982 (0.97–0.99)	0.022	0.98 (0.97–0.99)	0.028	–		–	
Numbers of HLA mismatch	1.097 (1.01–1.2)	0.036	1.09 (1–1.19)	0.049	1.01 (0.92–1.11)	0.81	1.01 (0.92–1.11)	0.85
Panel reactive antibody (max)	–		–		1.01 (1–1.02)	0.067	1.01 (1–1.02)	0.081
Donor: hypertension	–		–		1.31 (0.94–1.8)	0.112	1.35 (0.97–1.89)	0.077
Donor cytokine (log pg/ml)	1.43 (1.09–1.88)	0.01	1.5 (1–2.24)	0.05	1.36 (1.06–1.75)	0.016	1.45 (1–2.1)	0.052
High IL-6/CXCL10^a^	1.614 (1.08–2.4)			0.019	1.61 (1.12–2.32)			0.011
**Heart transplant**								
Donor age	1.03 (1–1.01)	0.079	1.03 (0.99–1.07)	0.081	1.02 (0.99–1.05)	0.177	1.02 (0.99–1.04)	0.18
Recipient age	1.01 (0.98–1.03)	0.76	1.00 (0.98–1.03)	0.79	–		–	
Waiting times for transplant (days)	–		–		1 (1–1.01)	0.095	1 (1–1.01)	0.095
Donor: cardiac arrest	–		–		1.88 (0.96–3.7)	0.065	1.87 (0.95–3.7)	0.068
Donor: cerebral vascular accident	3.36 (0.77–14.7)	0.11	3.75 (0.86–16.3)	0.078	2.13 (1.13–4.03)	0.019	2.16 (1.14–4.11)	0.018
Donor: trauma	1.16 (0.22–6.1)	0.86	1.3 (0.25–6.7)	0.76	–		–	
Donor cytokine (log pg/ml)	1.49 (0.83–2.68)	0.18	1.94 (0.82–4.6)	0.13	0.95 (0.59–1.53)	0.829	1.17 (0.61–2.27)	0.63
High IL-6/CXCL10^a^	2.15 (0.96–4.8)			0.064	1.52 (0.72–3.18)			0.28

For each cytokine, donor and recipient characteristics with p values < 0.05 in univariate analysis were included in the multivariate analysis.

^a^Inserted instead of donor cytokine.

## Discussion

To our knowledge, this is the largest prospective study examining whether the inflammatory status of the donor after brain death provides independent additional prediction of graft outcome among recipients followed according to standard clinical practice. Experiments in animal models have previously demonstrated the relation between brain death and the rapid infiltration of leukocyte populations in peripheral organs with intense upregulation of their associated products^14^. Concordantly, human studies have suggested that brain death of potential organ donors induces an inflammatory response mediated by IL-1b, IL-6, TNF alpha, CXCL1, CCL2, CCL5^39–41^ that could affect graft quality and function^42,43^. Although our study is not without limitations**,** it has generated valuable indications. First, we focused our analysis on two immunological mediators which have a common double advantage: to be extremely relevant for the immune response after transplantation and to be the target of drugs already available on the market (i.e., Tocilizumab, Sarilumab) or in advanced experimental clinical phases in humans (i.e., Eldelumab). IL-6 is critical for the progression of naïve B cells transforming into plasmablasts and mature plasma cells as well as shaping T cell immunity and is also responsible for activating the production of IL-17 signalling, inhibiting Treg function^44^. The chemokine CXCL10 is a potent chemoattractant for macrophages, dendritic cells, NK cells, and activated T cell and its level rapidly rises following organ reperfusion and during early rejection of the heart, kidney and liver^45^. Second, and equally important, we were able to define the correlation between IL-6 and CXCL10 levels of donor after brain death and their baseline characteristics. The evidence that both IL-6 and CXCL10 levels were significantly different from those in healthy subjects confirmed the detrimental effect of brain death in modifying the homeostasis of the immune system within a short time. These changes presumably occur secondary to the initial activity of catecholamine as well as circulatory cytokines originating from the injured brain^17–19^ Collectively, the donor characteristics explained a relatively low percentage of the variance (12–14%) in IL-6 and CXCL10 values, underling that brain death itself is probably the major driver of the “inflammatory signature”. Unfortunately, for many of the characteristics it is not possible to determine whether the relationship is of cause-effect. Despite this, some results are of great interest. For example, variables related to intensive care duration and hemodynamic instability, including the use of inotropes and vasopressors like norepinephrine, resulted generally associated with higher levels of circulating IL-6 and CXCL10. On the other side, not both the cytokines have the same relationship with baseline characteristics. Different causes of death appeared to determine different effects on measured cytokines with a relevant impact on CXCL10 but not IL-6 concentration. Kidney and liver function tests showed strong positive correlation with both circulating cytokines, while white blood cells showed a negative correlation. Taken as a whole these data showed that the activation of the brain-dead donor inflammatory response is very complex, and the mediators do not always respond in an identical way. Third, we were able to demonstrate the independent role of donor IL-6 and CXCL10 on graft outcome. This suggests that treatment aimed to reduce the donor inflammatory response could have impact on graft outcome. The identification of donor mediators acting as “master predictor” of outcome for all transplants is a “conditio sine qua non” to start to treat donors with drugs able to inhibit specific molecular pathways with the intention to reduce the inflammatory response in multiorgan donors and thereby improving organ survivals. Until now, this type of study has not yet been carried out for various reasons, including the difficulty of identifying the best targets for different organ grafts, the unavailability of measurable biomarkers and the difficulty of identifying a timing of treatment compatible with the explant procedures. Our results could fill some of these gaps. In fact, our data suggest a framework in which the inhibition of IL-6 and/or CXCL10 at the beginning of the donor observation period could be measured and the results used to improve the outcome of transplantation. The delay (kidney and heart) or the absence (liver) of functional recovery after transplantation was documented as being consistently associated with the donor inflammatory state. Concordantly, preventing the delay or lack of functional recovery of transplanted organs could represent the primary end-point of a study aimed at inhibiting donor IL-6/CXCL10. Of note, in the long-term follow up, factors more closely related to the specificity of individual organs could be influenced in different ways in each specific organ.

The findings of our study must be also seen in light of some limitations. First, we observed our cohort after a median follow-up of about 5 years and therefore the study lacks information of possible long-term effects of donor cytokines/chemokines on outcome. Second, deep data about the incidence of rejection episodes and the causes of graft failure were not always available. This makes difficult to understand whether donor factors have prediction value for specific causes of graft loss (i.e., cellular vs humoral rejection). Third, the cytokine levels were measured from the serum specimen sent for tissue typing, and at only that point. The time between blood sampling for circulating CXCL10 and IL-6 determination and organ procurement was quite homogenous in the different intensive care units (6–12 h), and this variability should not represent a major limitation for the study. Despite this, it is well known that inflammatory changes after brain death are dynamic and very heterogeneous, and we cannot exclude that significant changes can occur between blood sampling and organ procurement. On the other hand, the choice to use the serum specimen obtained at the beginning of the observation period was the only one possible, considering the number of intensive care units involved and of donors analyzed. Furthermore, in a pragmatic application perspective, it is the most standardizable observation point in organizational terms and would allow obtaining results in time for the selection of donors and recipients. Fourth, we did not included other donor types as controls, e.g., donor after cardiac death or living-related donors. During our study (2010–2012), transplants from donors after cardiac death were rare (less than 10) and, therefore, were not included in our analysis. On the other hand, living related organ donors were excluded from the analysis because they represent a highly selected population (to meet the living donation criteria) which does not obviously include heart donors.

In conclusion, this study was conducted to test the hypothesis that the inflammatory status of the heart beating multiorgan donor at the time of organ recovery provides independent incremental prediction of graft outcome among recipients followed according to standard clinical practice. The results confirmed the starting hypothesis. The characterization of the inflammatory signature may bring new therapeutic approaches in the transplant field. In fact, attenuating the donor inflammatory response before organ procurement may improve early and long-term outcomes after organ transplantation, and help maximize organ use from the available donor pool.

## Material and methods

### Study population and data sources

The study population consists of recipients who received organs from deceased individuals from whom organs were procured from January 1, 2010 to June 30, 2012 in The Nord Italia Transplant program (NITp) area. NITp is an inter‐regional transplant agency comprising six Italian regions: Lombardia, Liguria, Veneto, Friuli‐Venezia Giulia, Marche and the Autonomous Province of Trento. This area has 129 intensive care and 43 transplant units (15 for kidney transplantation, 5 for kidney and pancreas, 9 for liver, 6 for heart, 2 for heart and lung, 5 for lung and 1 for the intestine) for a population of 19 million inhabitants. NITp manages waiting lists, performs pre-transplant immunological tests, allocates organs, collects and analyses data (on recipients, organs and donors), organizes organ procurement, transport and transplant. A total of 1100 donors after brain death were considered during this period and their related recipients were prospectively included, obtaining a cohort made up of 2700 recipients with complete follow-up records. A non-diluted venous blood sample drawn from each donor at the procuring hospital was shipped to NITp central laboratory for crossmatch. The serum specimens were obtained in the participating intensive care units 6–12 h before procurement. An aliquot of 1 ml of serum was separated and stored at the Interregional Coordinating Center at − 80 °C for the measurements of inflammatory/immunological factors. The mean time from sampling and freezing was 3.67 h and during this time the sample was conserved at 4 °C. There was no evidence of significant change in IL-6 and CXCL10 levels related to the time from sample collection until freezing. Information on donor demographics and medical characteristics and the disposition of each organ that could potentially be used for transplantation therapy was obtained from the deceased donor registry data maintained by the NITp. All donor data were abstracted from the donor medical records and entered on standardized NITp donor data collection form by the transplant coordinator at the procuring hospital. Donor characteristics used in our analysis can be found in Table S1. Serum from 55 healthy subjects was used as control group (M/F: 22/33; age: 49.5 ± 15; BMI: 21.6 ± 3.2). The study was supported and approved by The Italian National Transplant Centre (CNT) and by San Raffaele IRB. All experiments on human subjects were conducted in accordance with the Declaration of Helsinki and, when appropriate, all procedures were carried out with the adequate understanding and consent of the subjects (i.e., consent for organ donation according to Italian law). Informed consent for cytokine assays on unused serum drawn for crossmatch was waived by the Comitato Etico Ospedale San Raffaele for brain death donor because it was impossible to ask incapacitated patients.

### Measurement of humoral inflammatory/immunological factors

For the study we used a bead-based assay based on Luminex technology (Bio-Rad, Milan, Italy), that allowed to measure both CXCL10 and IL-6 using a low volume of serum (50 µl). To minimize inter-assay variation, donor sera was assayed at the end of the study using the same commercial lot. Appropriate pool of sera was used to estimate intra-assay and inter-assay coefficient of variation.

### Outcomes and follow-up

In Italy, organ donation and transplantation activities are coordinated by law by the CNT that, in collaboration with regional and interregional coordinating Centers, ensures the quality and traceability of the entire process all over the national territory. To this purpose, all transplants performed in Italy are recorded on the Transplant Information System (SIT). Transplant activity data registered in SIT were used to evaluate graft failure and recipient survival. Graft survival was defined as time from transplant to graft failure, censoring for death with a functioning graft and grafts still functioning at time of analysis. Patient survival was defined as time from transplant to patient death, censoring for patients still alive at time of analysis. Recipient at immunological risk included patients with high rate of antibodies (> 50% antibodies against panel), recipients who had lost their first graft due to early rejection, cross match positive or HLA incompatibility according to immunology service center criterion^46^. Immediate Graft Function (IGF) was defined as the absence of early allograft dysfunction as previously defined after kidney^47^, liver^48^ and heart transplantation^49^. Kidney primary non function (PNF) was defined as the absence of a decrease in serum creatinine levels, which resulted in the need for dialysis 3 months after transplant. Acute and chronic kidney rejection were defined as biopsy proven or clinically evident rejection. Liver PNF was defined as graft failure resulting in death or retransplantation within 30 days of the index transplant when other causes of graft failure were excluded, i.e., vascular thrombosis, rejection, or recurrent disease. Acute and chronic liver rejection was defined according to the International Consensus Document on Terminology of Hepatic Allograft Rejection^50^. Heart Primary Graft Failure (PGF) was defined as previously reported^49^. Acute and chronic heart rejection was defined according to the International Consensus Document on nomenclature in the diagnosis of heart rejection^51^. NITK3 is an allocation algorithm established in 1997, which aims at ensuring quality, equity, transparency and traceability during all the phases of the allocation decision-making process^52^. NITK3 has been set up by the NITp Working Group on the basis of biological, medical and administrative criteria and it is periodically reviewed after the analysis of transplant results^52^.

### Statistical analysis

Data are presented as mean ± standard deviation (SD) or median and interquartile range (IQR), according to their distribution. Class variables are given as numbers of individuals in percentages. Variables with a normal distribution were compared with one-way unpaired Student’s t test. Variables with a non-normal distribution were compared with Mann–Whitney U test. Categorical variables were compared using the chi-squared test or Fisher’s exact test as appropriate. Spearman correlation was used to study associations of continuous variables. Graft or patient survivals were estimated according to Kaplan–Meier. Association between variables and outcomes or survivals was assessed by Logistic or Cox proportional-hazard regression, respectively. For the regression models, cytokines concentrations were used as continuous variables after Log transformation or as preplanned binary variables: 0 if the value was ≤ 66th percentiles, 1 if the value was > 66th percentiles defining “high IL-6” category for concentration > 563 pg/ml (66th percentile), and “high CXCL10” category for concentration > 1748 pg/ml (66th percentile). The multivariate analysis was performed using variables significant at the p < 0.1 in the univariate analysis. All statistical analyses were performed using the SPSS statistical software, version 13.0 (SPSS Inc, Chicago, IL, USA).

## Supplementary Information

Supplementary Information

## Abbreviations

CXCL10: C-X-C motif chemokine 10
CMV: Cytomegalovirus
ICU: Intensive care unit
IGF: Immediate graft function
IL-6: Interleukin 6
IQR: Interquartile range
NITp: The Nord Italia Transplant program
PRA: Panel-reactive antibody
WBC: White blood cells
