# Supplementary material for: Circulating CXCL10 and IL-6 in solid organ donors after brain death predict graft outcomes
Source: Sci Rep. 2021 Mar 23;11:6624. doi: 10.1038/s41598-021-86085-6 (PMC7988181; doi:10.1038/s41598-021-86085-6)
Supplement: Supplementary file 1 — Supplementary Information [file 41598_2021_86085_MOESM1_ESM.docx]

**Circulating CXCL10 and IL-6 in solid organ donors after brain death predict graft outcomes**

Lorenzo Piemonti, MD; Valeria Sordi, PhD; Silvia Pellegrini, PhD; Giulia Maria Scotti, PhD; Marina Scavini, MD; Viviana Sioli, MD; Andrea Gianelli Castiglione, MD; Massimo Cardillo*,* MD.

**Supplementary tables**

**Supplementary Table 1.** Descriptive data for recovered organ donor January 2010 to June 2012 (n=1100).

| Variable | Value | Missing data | Description |
| --- | --- | --- | --- |
| Age | 57.5±17 | 0 | Donor age in years |
| Female | 46.5% (512/588) | 0 | Dichotomous 0–1 variable: 1 if the donor is female, 0 otherwise |
| BMI | 25.6±4.12 | 0 | Donor BMI |
| Blood type A | 44.5% (490/1100) | 0 | Dichotomous 0–1 variable: 1 if the donor is blood type A, 0 otherwise |
| Blood type B | 43.1% (474/1100) | 0 | Dichotomous 0–1 variable: 1 if the donor is blood type B, 0 otherwise |
| Blood type AB | 3.7% (41/1100) | 0 | Dichotomous 0–1 variable: 1 if the donor is blood type AB, 0 otherwise |
| Blood type 0 | 8.6% (95/1100) | 0 | Dichotomous 0–1 variable: 1 if the donor is blood type 0, 0 otherwise |
| Cause of death: cardiovascular accident | 63.5% (699/1100) | 0 | Dichotomous 0–1 variable: 1 if the donor's death was due to a cardiovascular accident, 0 otherwise |
| Cause of death: trauma | 20.1% (221/1100) | 0 | Dichotomous 0–1 variable: 1 if the donor's death was due to a traumatic event, 0 otherwise |
| Cause of death: anoxic event | 9.5% (104/1100) | 0 | Dichotomous 0–1 variable: 1 if the donor's death was due to an anoxic event, 0 otherwise |
| Cause of death: primary brain tumor | 0.7% (8/1100) | 0 | Dichotomous 0–1 variable: 1 if the donor's death was due to a primary brain tumor, 0 otherwise |
| Cause of death: others | 6.2% (68/1100) | 0 | Dichotomous 0–1 variable: 1 if the donor's death was due to a cause of death that was not from the above, 0 otherwise |
| Suitability of organ donor: standard risk | 68.8% (757/1100) | 0 | Dichotomous 0–1 variable: 1 if the evaluation process does not identify risk factors for any transmissible disease, 0 otherwise |
| Lengths of stay in Intensive Care Unit | 4.1±4.7 | 0.2% | Time, in days, from admission in ICU to recovery |
| Any inotrope administered | 69.1% (760/897) | 18.5% | Dichotomous 0–1 variable: 1 if any inotrope was administered prior to recovery, 0 otherwise |
| Two or more inotropes running at time of recovery | 15.2% (167/897) | 18.5% | Dichotomous 0–1 variable: 1 if two or more inotropes were being administered at time of recovery, 0 otherwise |
| Norepinephrine administered | 56.9% (511/897) | 18.5% | Dichotomous 0–1 variable: 1 if norepinephrine was administered prior to recovery, 0 otherwise |
| Dopamine administered | 30.4% (334/897) | 18.5% | Dichotomous 0–1 variable: 1 if dopamine was administered prior to recovery, 0 otherwise |
| Cardiac arrest | 21.9% (194/887) | 19.4% | Dichotomous 0–1 variable: 1 if the donor had arrested, 0 otherwise |
| Proteinuria during this admission | 38.3% (250/653) | 40.6% | Dichotomous 0–1 variable: 1 if the donor had the presence of proteinuria prior to recovery, 0 otherwise |
| History of diabetes | 14.9% (131/881) | 19.9% | Dichotomous 0–1 variable: 1 if the donor had a history of diabetes (type I or II), 0 otherwise |
| History of hypertension | 51.5% (478/928) | 15.6% | Dichotomous 0–1 variable: 1 if the donor had a history of hypertension controlled by medication, 0 otherwise |
| History of cardiomyopathy | 42.2% (242/574) | 47.8% | Dichotomous 0–1 variable: 1 if the donor had a history of cardiomyopathy, 0 otherwise |
| Donor cancer history | 6.5% (55/839) | 23.7% | Dichotomous 0–1 variable: 1 if the donor had a history of cancer (any type), 0 otherwise |
| History of smoking | 35.3% (304/860) | 21.8% | Dichotomous 0–1 variable: 1 if the donor had a history of smoking (any type), 0 otherwise |
| History of ethilism | 70.4% (59/838) | 23.8% | Dichotomous 0–1 variable: 1 if the donor had a history of ethilism (any type), 0 otherwise |
| Final white blood cells (WBC) | 12,414±5,312 | 28.3% | Final recorded WBC (cell/mm3) |
| Final Haemoglobin (Hb) | 10.9±1.9 | 29.2% | Final recorded Hb (g/dL) |
| Final creatinine | 1.12±0.97 | 26.7% | Final recorded creatinine (mg/dL) |
| Final blood urea nitrogen | 39.7±28.9 | 37.7% | Final recorded urea nitrogen (mg/dL) |
| Final aspartate aminotransferase (AST) | 66.4±137 | 29% | Final recorded blood AST (U/L) |
| Final Alanine Aminotransferase (ALT) | 56.2±109 | 28% | Final recorded blood ALT (U/L) |
| Total bilirubin | 1.04±2.22 | 30.72% | Final recorded total bilirubin (mg/dL) |
| Final gamma-glutamyl transferase (GGT) | 68.1±105 | 34% | Final recorded GGT (U/L) |
| Final amylase | 208.8±372 | 42.6% | Final recorded amylase (U/L) |
| Final international normalized ratio (INR) | 1.28±0.37 | 34% | Final recorded INR |
| Donor urinary infection | 15.9% (133/833) | 24.3% | Dichotomous 0–1 variable: 1 if the donor had a urinary infection (positive urinary culture) during this admission, 0 otherwise |
| Donor blood infection | 9.3% (82/878) | 20.2% | Dichotomous 0–1 variable: 1 if the donor had a blood infection (positive blood-culture) during this admission, 0 otherwise |
| Donor pulmonary infection | 50.7% (427/842) | 23.5% | Dichotomous 0–1 variable: 1 if the donor had a pulmonary infection (positive Broncho-alveolar lavage) during this admission, 0 otherwise |
| Donor hepatitis B core antibody (HBcAb) positive | 19.4% (213/1100) | 0 | Dichotomous 0–1 variable: 1 if the donor's serology testing was positive for HBcAb, 0 otherwise |
| Donor hepatitis C virus (HCV) positive | 2.1% (23/1100) | 0 | Dichotomous 0–1 variable: 1 if the donor's serology testing was positive for hepatitis C, 0 otherwise |
| Donor Epstein Barr nuclear antigen antibody (EBV-EBNA IgG) positive | 91.8% (962/1048) | 4.7% | Dichotomous 0–1 variable: 1 if the donor's serology testing was positive for EBV-EBNA IgG, 0 otherwise |
| Donor Epstein Barr viral capsid antigen antibody (EBV-VCA IgG) positive | 93% (1010/1086) | 1.3% | Dichotomous 0–1 variable: 1 if the donor's serology testing was positive for EBV-VCA IgG, 0 otherwise |
| Donor Cytomegalovirus antibody (CMV-IgG) positive | 85.5% (930/1088) | 1.1% | Dichotomous 0–1 variable: 1 if the donor's serology testing was positive for CMV-IgG, 0 otherwise |
| Donor Cytomegalovirus antibody (CMV-IgM) positive | 0.2% (2/1092) | 0.7% | Dichotomous 0–1 variable: 1 if the donor's serology testing was positive for CMV-IgM, 0 otherwise |
| Donor Herpes Simplex Virus-1 antibody (HSV-1 IgG) positive | 92.6% (984/1062) | 3.5% | Dichotomous 0–1 variable: 1 if the donor's serology testing was positive for HSV-1 IgG, 0 otherwise |
| Donor Herpes Simplex Virus-2 antibody (HSV-2 IgG) positive | 8.4% (91/1086) | 1.3% | Dichotomous 0–1 variable: 1 if the donor's serology testing was positive for HSV-2 IgG, 0 otherwise |
| Donor Toxoplasma gondii antibody (Toxo-IgG) positive | 58.2% (624/1072) | 2.5% | Dichotomous 0–1 variable: 1 if the donor's serology testing was positive for Toxo-IgG, 0 otherwise |
| Donor Toxoplasma gondii antibody (Toxo-IgM) positive | 0.9% (10/1085) | 1.4% | Dichotomous 0–1 variable: 1 if the donor's serology testing was positive for Toxo-IgM, 0 otherwise |
| Donor Treponema Pallidum Haemagllutination Assay (TPHA) positive | 0.54% (6/1098) | 0.2% | Dichotomous 0–1 variable: 1 if the donor's serology testing was positive for TPHA, 0 otherwise |
| Donor Varicella Zoster Virus antibody (VZV-IgG) positive | 92.3% (1002/1085) | 1.4% | Dichotomous 0–1 variable: 1 if the donor's serology testing was positive for VZV-IgG, 0 otherwise |

**Supplemental table 2. Organ transplantation by regions**

| **Region** | Center | Kidney | | | | | | | liver | | | | | | Heart | | | Lung | | pancreas |
| --- | --- | --- | --- | --- | --- | --- | --- | --- | --- | --- | --- | --- | --- | --- | --- | --- | --- | --- | --- | --- |
|  |  | S | D | +P | +L | +L  (RL) | +L  (LL) | +P  +L(RL) | W | RL | RL+P | LL | +P | +P  +L(D) |  | +K | +L(D) | S | D |  |
| Lombardia | 1 | 69 | 16 | 2 | 3 | 0 | 1 | 1 | 127 | 9 | 2 | 34 | 1 | 1 | 46 | 0 | 0 | 6 | 17 | 0 |
|  | 2 | 123 | 0 | 0 | 0 | 0 | 0 | 0 | 0 | 0 | 0 | 0 | 0 | 0 | 0 | 0 | 0 | 0 | 0 | 0 |
|  | 3 | 114 | 12 | 7 | 6 | 2 | 0 | 0 | 152 | 8 | 0 | 0 | 0 | 0 | 51 | 1 | 0 | 2 | 4 | 2 |
|  | 4 | 92 | 0 | 0 | 2 | 0 | 0 | 0 | 92 | 8 | 0 | 0 | 0 | 0 | 0 | 0 | 0 | 8 | 12 | 0 |
|  | 5 | 12 | 0 | 0 | 1 | 0 | 0 | 0 | 0 | 0 | 0 | 0 | 0 | 0 | 0 | 0 | 0 | 0 | 0 | 0 |
|  | 6 | 66 | 8 | 14 | 0 | 0 | 0 | 0 | 0 | 0 | 0 | 0 | 0 | 0 | 0 | 0 | 0 | 0 | 0 | 11 |
|  | 7 | 0 | 0 | 0 | 0 | 0 | 0 | 0 | 82 | 3 | 0 | 0 | 0 | 0 | 0 | 0 | 0 | 0 | 0 | 0 |
|  | 8 | 71 | 0 | 0 | 0 | 0 | 0 | 0 | 0 | 0 | 0 | 0 | 0 | 0 | 48 | 0 | 3 | 8 | 10 | 0 |
|  | 9 | 109 | 2 | 0 | 0 | 0 | 0 | 0 | 0 | 0 | 0 | 0 | 0 | 0 | 0 | 0 | 0 | 0 | 0 | 0 |
|  | *Tot* | *656* | *38* | *23* | *12* | *2* | *1* | *1* | *453* | *28* | *2* | *34* | *1* | *0* | *145* | *1* | *3* | *24* | *43* | *13* |
| Veneto | 10 | 106 | 34 | 17 | 2 | 0 | 0 | 0 | 158 | 12 | 0 | 9 | 0 | 0 | 44 | 0 | 0 | 11 | 39 | 3 |
|  | 11 | 22 | 0 | 0 | 0 | 0 | 0 | 0 | 0 | 0 | 0 | 0 | 0 | 0 | 0 | 0 | 0 | 0 | 0 | 0 |
|  | 12 | 105 | 8 | 0 | 0 | 0 | 0 | 0 | 0 | 0 | 0 | 0 | 0 | 0 | 0 | 0 | 0 | 0 | 0 | 0 |
|  | 13 | 52 | 10 | 0 | 0 | 0 | 0 | 0 | 0 | 0 | 0 | 0 | 0 | 0 | 0 | 0 | 0 | 0 | 0 | 0 |
|  | 14 | 121 | 47 | *0* | 0 | 0 | 0 | 0 | 49 | 5 | *0* | 0 | *0* | *0* | *30* | *0* | *0* | *0* | *0* | *0* |
|  | *Tot* | *406* | *99* | *17* | *2* | *0* | *0* | *0* | *207* | *17* | *0* | *9* | *0* | *0* | *74* | *0* | *0* | *11* | *39* | *3* |
| Friuli‐Venezia Giulia | 15 | 0 | 0 | 0 | 0 |  |  |  | 45 | 7 |  | 0 |  |  | 0 | 0 | 0 | 0 | 0 | 0 |
|  | 16 | 94 | 4 | 3 | 0 | 0 | 0 | 0 | 2 | 0 | 0 | 0 | 0 | 0 | 53 | 2 | 0 | 0 | 0 | 0 |
|  | *Tot* | *94* | *4* | *3* | *0* | *0* | *0* | *0* | *48* | *7* | *0* | *0* | *0* | *0* | *53* | *2* | *0* | *0* | *0* | *0* |
| Marche | 17 | 86 | 0 | 0 | 0 | 0 | 0 | 0 | 88 | 2 | 0 | 0 | 0 | 0 | 0 | 0 | 0 | 0 | 0 | 0 |
|  | *Tot* | *86* | *0* | *0* | *0* | *0* | *0* | *0* | *88* | *2* | *0* | *0* | *0* | *0* | *0* | *0* | *0* | *0* | *0* | *0* |
| Liguria | 18 | 6 | 0 | 0 | 0 | 0 | 0 | 0 | 0 | 0 | 0 | 0 | 0 | 0 | 0 | 0 | 0 | 0 | 0 | 0 |
|  | 19 | 21 | 2 | 3 | 1 | 0 | 0 | 0 | 14 | 2 | 0 | 0 | 0 | 0 | 0 | 0 | 0 | 0 | 0 | 0 |
|  | 20 | 8 | 0 | 0 | 0 | 0 | 0 | 0 | 0 | 0 | 0 | 0 | 0 | 0 | 0 | 0 | 0 | 0 | 0 | 0 |
|  | 21 | 48 | 7 | 0 | 0 | 0 | 0 | 0 | 5 | 0 | 0 | 0 | 0 | 0 | 0 | 0 | 0 | 0 | 0 | 0 |
|  | *Tot* | 83 | 9 | 3 | 1 | 0 | 0 | 0 | 19 | 2 | 0 | 0 | 0 | 0 | 0 | 0 | 0 | 0 | 0 | 0 |
| **TOT** |  | **1325** | **150** | **46** | **15** | **2** | **1** | **1** | **815** | **56** | **2** | **43** | **1** | **1** | **272** | **3** | **3** | **35** | **82** | **16** |

S= single; D= double; W= whole; RL= right lobe; LL= left lobe; P= pancreas; K=kidney; L= lung

**Supplementary table 3. Cox proportional hazard models of the predictors of graft failure and recipient mortality by univariate analysis**

|  | Graft failure | | | | | | Recipient death | | | | | |
| --- | --- | --- | --- | --- | --- | --- | --- | --- | --- | --- | --- | --- |
|  | Kidney | | Liver | | Heart | | Kidney | | Liver | | Heart | |
|  | HR  (95% CI) | p | HR  (95% CI) | p | HR  (95% CI) | p | HR  (95% CI) | P | HR  (95% CI) | p | HR  (95% CI | P |
| Recipient Age | 1  (.98-1.01) | .69 | .98  (.97-1) | ***.02*** | 1.02  (.99-1.05) | .18 |  |  |  |  |  |  |
| Age classes (0-39aa; 40-59aa; >60aa) | 1.06  (.82-1.01) | .64 | 0.81  (.59-1.12) | .20 | 1.66  (.98-2.82) | .06 |  |  |  |  |  |  |
| Donor Age | 1.01  (1-1.03) | ***.03*** | 1  (.99-1.01) | .99 | 1.05  (1.02-1.09) | ***.001*** | 1.02  (1-1.04) | ***.05*** | 1.01  (1-1.02) | .22 | 1.02  (1-1.05) | ***.03*** |
| Age classes (0-39aa; 40-59aa; >60aa) | 1.51  (1.16-1.96) | ***.001*** | .99  (.77-1.28) | .94 | 2.63  (1.53-4.5) | ***<.001*** | 1.37  (.93-2.02) | .12 | 1.03  (.84-1.26) | .81 | 1.62  (1.09-2.4) | ***.02*** |
| Recipient female gender | .82  (.56-1.19) | .29 | .97  (.77-1.2) | .75 | 1.06  (.71-1.58) | .79 |  |  |  |  |  |  |
| Donor female gender | .79  (.55-1.12) | .19 | .94  (.66-1.35) | .75 | 1.26  (.63-2.53) | .51 | 1.2  (.81-1.76) | .37 | 0.93  (.7-1.23) | .60 | .76  (.45-1.29) | .32 |
| Gender match | 1.05  (.74-1.49) | .78 | 1.07  (.74-1.54) | .72 | 1.25  (.58-2.71) | .57 | 1.08  (.73-1.59) | .70 | 1.05  (.79-1.4) | .71 | 1.19  (.7-2.02) | .51 |
| HLA-A mismatch | .87  (.67-1.13) | .30 | 1.24  (.97-1.58) | .08 | .64  (.36-1.14) | .13 | .93  (.69-1.26) | .64 | 1.22  (1-1.49) | ***.05*** | .91  (.64-1.29) | .58 |
| HLA-B mismatch | .87  (.67-1.13) | .29 | 1.27  (1.02-1.58) | ***.03*** | .75  (.47-1.21) | .24 | .95  (.71-1.27) | .71 | 1.17  (.97-1.4) | .18 | .93  (.68-1.28) | .93 |
| HLA-DR mismatch | 1.1  (.84-1.43) | .50 | 1.28  (.99-1.65) | .06 | .63  (.33-1.2) | .16 | .9  (.67-1.21) | .50 | 1.27  (1.03-1.57) | ***.03*** | 1  (.69-1.44) | .99 |
| Numbers of HLA mismatch | .95  (.85-1.09) | .47 | 1.1  (1.01-1.2) | ***.04*** | .86  (.71-1.05) | .14 | .94  (.81-1.09) | .44 | 1.08  (1-1.16) | ***.04*** | .98  (.86-1.11) | .71 |
| Panel Reactive Antibody (max) | 1.11  (1.03-1.19) | ***.01*** | 1.01  (.09-1.02) | .36 | .89  (.67-1.18) | .41 | 1.01  (1-1.02) | .06 | 1.01  (1-1.02) | ***.03*** | .85  (.64-1.14) | .28 |
| Panel Reactive Antibody (yes/no) | 1.4  (.92-2.12) | .12 | 1.18  (.65-2.14) | .59 | .05  (0-17.86) | .31 | 1.34  (.82-2.19) | .25 | 1.22  (.76-1.97) | .41 | .23  (.03-1.73) | .16 |
| Immunologic risk (yes/no) | 1.64  (1.07-2.5) | ***.02*** |  |  |  |  | 1.58  (.93-2.68) | .09 |  |  |  |  |
| Waiting times for transplant (days) | 1  (1-1) | .77 | 1  (1-1) | .19 | 1  (1-1) | .81 | 1  (1-1) | .17 | 1  (1-1) | .77 | 1  (1-1) | ***.03*** |
| Waiting time classes (<3aa; 3-10aa; >10aa) | 1.2  (.85-1.69) | .31 | 1.21  (.45-2.28) | .71 | 1.33  (.41-4.38) | .64 | 1.22  (.82-1.81) | .33 | 1.12  (.52.53) | .79 | 1.88  (.92-3.82) | .08 |
| NITK3 | .87  (.74-1.01) | .06 |  |  |  |  | 0.87  (.73-1.05) | .14 |  |  |  |  |
| Cold ischemia time (min) | 1.04  (1.01-1.06) | ***.01*** | .99  (.97-1.01) | .41 | 1  (1-1.01) | .74 | 1.02  (.98-1.06) | .33 | 1  (.99-1) | .29 | 1  (1-1) | .67 |
| Donor: ICU stay (days) | .99  (.86-1.03) | .72 | 1.02  (.99-1.06) | .20 | 1.01  (.95-1.07) | .84 | .98  (.94-1.03) | .40 | 1.02  (.99-1.05) | .27 | .99  (.94-1.05) | .72 |
| Donor: cardiac arrest | 1  (.64-1.57) | .99 | .86  (.53-1.41) | .55 | 1.01  (.35 – 2.93) | .99 | 1.26  (.77-2.04) | .36 | 0.87  (.59-1.28) | .48 | 1.79  (.95-3.36) | .07 |
| Donor: diabetes | .84  (.37-1.91) | .67 | 1.11  (.63-1.95) | .72 | 1.62  (.22-11.99) | .64 | .8  (.35-1.86) | .61 | 1.01  (.63-1.62) | .97 | 0.55  (.08-4.01) | .55 |
| Donor: hypertension | 1.39  (.96-2.03) | .08 | 1.07  (.73-1.57) | .75 | 1.91  (.83-4.4) | .13 | 1.45  (.95-2.21) | .09 | 1.34  (.98-1.83) | .07 | .93  (.48-1.83) | .84 |
| Donor: hypotension | 1.01  (.66-1.55) | .96 | .87  (.54-1.39) | .56 | .7  (.26 – 1.88) | .48 | 1.15  (.7-1.89) | .57 | .89  (.62-1.28) | .52 | 1.04  (.55-1.99) | .91 |
| Donor: cerebral vascular accident | 1.38  (.96-1.99) | .08 | .85  (.59-1.22) | .37 | 4.38  (1.9-10.14) | ***,00*** | .8  (.54-1.2) | .29 | 1.11  (.83-1.5) | .47 | 2.08  (1.24-3.49) | ***.01*** |
| Donor: trauma | .77  (.49-1.19) | .24 | 1.31  (.86-2.01) | .21 | .32  (.12-.82) | ***.02*** | 1.09  (.67-1.76) | .73 | 1.22  (.87-1.71) | .25 | 0.65  (.37-1.14) | .13 |
| Donor: inotrope administered | .63  (.45-.92) | ***.02*** | 1.32  (.95- 1.85) | .10 | 1.64  (.78-3.45) | .20 | 1.12  (.78-1.6) | .55 | .99  (.76-1.29) | .92 | 1.44  (.87-2.38) | .16 |
| IL-6 (log pg/ml) | 1  (.76-1.31) | .98 | 1.4  (1.07-1.85) | ***.02*** | 1.5  (.85-2.64) | .16 | 1.24  (.93-1.65) | .15 | 1.34  (1.08-1.66) | ***.01*** | 1.11  (.74-1.66) | .62 |
| High IL-6 (highest tertile) | 1.1  (.76-1.58) | .62 | 1.38  (.96-1.99) | .08 | 1.28  (.62-2.65) | .51 | 1.42  (.96-2.11) | .08 | 1.36  (1.03-1.81) | ***.03*** | 1  (.59-1.72) | .97 |
| CXCL10 (log pg/ml) | 1  (.67-1.5) | .99 | 1.5  (1-2.24) | ***.05*** | 1.66  (.74-3,75) | .22 | 1.09  (.7-1.76) | .70 | 1.28  (.93-1.76) | .13 | 1.23  (.71-2.16) | .46 |
| High CXCL10 (highest tertile) | 1.08  (.75-1.55) | .67 | 1.33  (.92-1.91) | .13 | 1.19  (.56-2.51) | .65 | 1.25  (.84-1.86) | .27 | 1.33  (1-1.76) | ***.05*** | 1.17  (.69-1.96) | .56 |
| High IL-6/high CXCL10 | 1.23  (.82-1.86) | .32 | 1.6  (1.07-2.39) | ***.02*** | 2.04  (.91-4.53) | .08 | 1.74  (1.14-2.64) | ***.01*** | 1.6  (1.17-2.19) | ***.001*** | 1.88  (1.04-3.41) | ***.04*** |

**
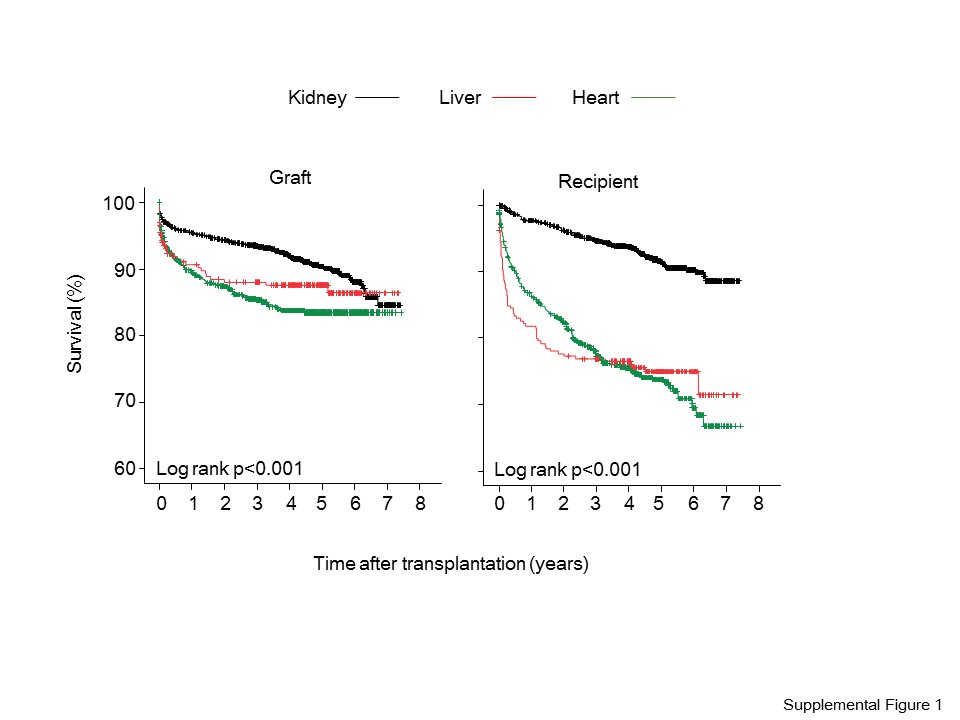
**

**Supplemental Figure 1.** Kaplan–Meier graft and recipient survival curves according to organ transplanted.

**
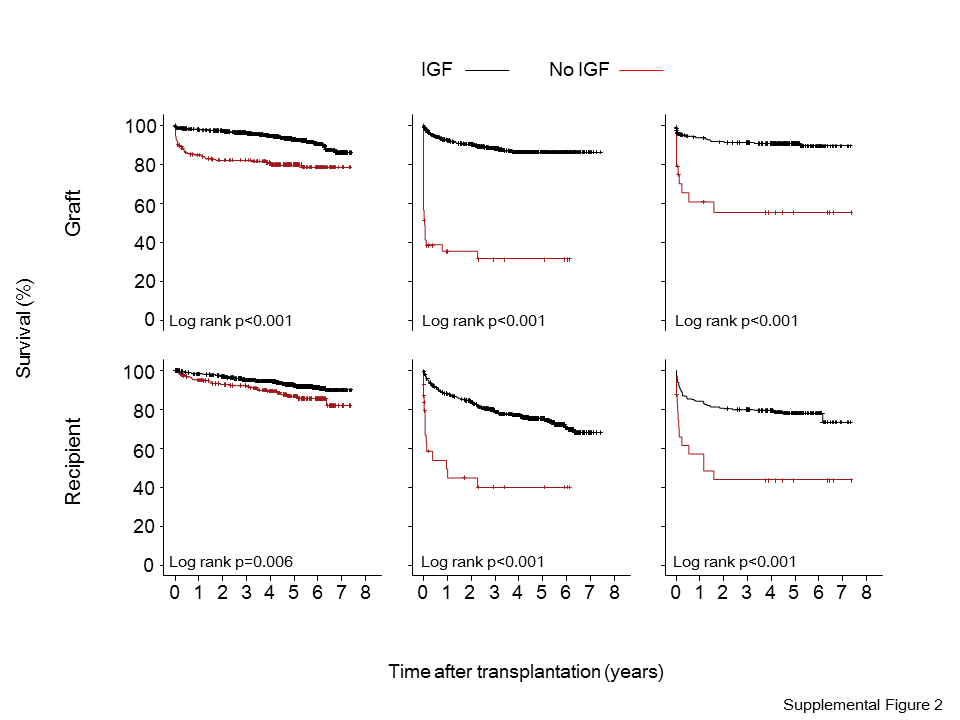
Supplementary Figure 2.** Kaplan–Meier graft and recipient survival curves according to Immediate Graft Function (IGF).
